# High-Performance γ-Al_2_O_3_ Multichannel Tube-Type Tight Ultrafiltration Membrane Using a Modified Sol-Gel Method

**DOI:** 10.3390/membranes13040405

**Published:** 2023-04-03

**Authors:** Danyal Naseer, Jang-Hoon Ha, Jongman Lee, Hong Joo Lee, In-Hyuck Song

**Affiliations:** 1 Ceramic Materials Division, Korea Institute of Materials Science (KIMS), 797 Changwon-daero, Changwon-si 51508, Republic of Korea; 2 Department of Advanced Materials Engineering, University of Science and Technology (UST), 217 Gajeong-ro, Daejeon 34113, Republic of Korea

**Keywords:** γ-Al_2_O_3_ membranes, mesoporous, sol-gel method, multichannel, high permeability

## Abstract

We introduced a modified sol-gel method using polyvinyl alcohol (PVA) as an additive to improve the permeability of γ-Al_2_O_3_ membranes by minimizing the thickness of the selective layer and maximizing the porosity. First, the analysis revealed that the thickness of γ-Al_2_O_3_ decreased as the concentration of PVA increased in the boehmite sol. Second, the properties of the γ-Al_2_O_3_ mesoporous membranes were greatly influenced by the modified route (method B) compared to the conventional route (method A). The results showed that the porosity and surface area of the γ-Al_2_O_3_ membrane increased, and the tortuosity decreased considerably using method B. This effect was attributed to the adsorption of PVA molecules on the surface of the boehmite particles, which depended on the synthesis route. The experimentally determined pure water permeability trend and the Hagen–Poiseuille mathematical model confirmed that the modified method improved the performance of the γ-Al_2_O_3_ membrane. Finally, the γ-Al_2_O_3_ membrane fabricated via a modified sol-gel method with a pore size of 2.7 nm (MWCO = 5300 Da) exhibited a pure water permeability of over 18 LMH/bar, which is three times higher than that of the γ-Al_2_O_3_ membrane prepared using the conventional method.

## 1. Introduction

Recently, the demand for resource recovery from harsh industrial wastewater has increased due to resource depletion and the need to achieve net-zero carbon emissions. In this regard, ultrafiltration (UF) membranes have been widely studied and utilized in various fields, including the recovery of dyes from industrial wastewater, cooling water for turbines, and the pretreatment for reverse osmosis. The molecular weight cut-off (MWCO) of tight UF membranes is 1000–10,000 Dalton (Da) [1]. Ceramic UF membranes with high tunability, stability in harsh chemical environments over a wide temperature range, and resistance to organic matter [2,3], are considered to have the most potential for wastewater treatment. Alumina (Al_2_O_3_) is a widely used ceramic material for membrane applications owing to its advantages such as high-temperature filtration [4] and hydrophilic nature, which mitigates fouling [5].

The most efficient routes for ceramic fabrication include sol-gel, dip-coating, and sintering [6]. The sol-gel process is considered to be the primary technique for fabricating ceramic mesoporous membranes. Colloidal sols consist mainly of precursors, hydrolysis agents, and additives, including peptizing agents and organic polymers [7,8,9].

Mesoporous γ-Al_2_O_3_ membranes were the first, and are currently the most widely investigated membranes for UF applications [10]. Mesoporous γ-Al_2_O_3_ membranes with an average pore size of 3–5 nm have been reported in the literature [11,12,13,14,15] as presented in Table 1, and they have been used as an intermediate layer for top nanofiltration coatings [16]. Nanofiltration γ-Al_2_O_3_ membranes with a mean pore size of 1–2 nm have also been developed [17,18]. However, the permeability of these membranes is not sufficient for industrial applications. The pure water permeability is generally reported to be less than 10 LMH/bar. One effective approach to fabricating γ-Al_2_O_3_ membranes with high permeability is to modify their morphological characteristics, including the surface area and porosity. These characteristics mainly depend on the synthesis conditions and the incorporation of additives [19,20,21].

In our previous research [22], we investigated the effect of acetic acid as a peptizing agent on the formation of mesoporous α-Al_2_O_3_ membranes. We optimized the peptization step to produce boehmite sol with a smaller particle size, which mitigated the growth of particles during the transformation from the γ- to the α-phase. The α-Al_2_O_3_ membrane with the smallest mean pore size of 12.5 nm (MWCO = 163 kDa) was obtained by synthesizing boehmite sol at pH 3.5 with a 24 h peptization time.

In this study, we investigated the effect of organic additives on the characteristics of γ-Al_2_O_3_ membranes. To date, organic polymers, including polyvinyl alcohol (PVA), cellulosic compounds, and polyglycols, have been added as binders in boehmite sol to prevent crack generation during thermal treatment [23]. However, the effect of organic additives on the morphological properties of membranes synthesized using boehmite sol remains unclear. 

PVA is the most frequently used additive for fabricating defect-free and stable Al_2_O_3_ membranes [24,25]. In the conventional sol-gel method, PVA is added to the sol after peptization. In this study, we introduce a modified sol-gel method using PVA to improve the permeability of a γ-Al_2_O_3_ tight UF membrane by minimizing the thickness of the selective layer and maximizing porosity. Research has been conducted on the development of unsupported Al_2_O_3_ membranes by altering the order in which PVA is added during the sol-gel process [26]. However, the effect of the interaction between boehmite particles and PVA molecules on the filtration properties has not been fully investigated. Therefore, further research into the synthesis of γ-Al_2_O_3_ membranes focusing on the interaction with PVA is required.

This study aimed to investigate the effect of PVA addition on the properties of γ-Al_2_O_3_ membranes. Emphasis was placed on the comparison of the membrane morphology and permeability before and after the peptization step as a function of the amount of PVA added.

## 2. Materials and Methods

### 2.1. Synthesis of Boehmite Sol

The synthesis of the boehmite sol involved several steps. Aluminum tri-sec-butoxide (ASB, 97%, Sigma-Aldrich, Darmstadt, Germany) was used as the precursor. Ethyl alcohol (C_2_H_6_O, 99.9%, Samchun Chemicals, Seoul, South Korea) was used as a solvent, deionized (DI) water as a hydrolysis agent, and acetic acid (CH_3_CO_2_H, ≥99.7%, Sigma Aldrich, Darmstadt, Germany) as a peptizing agent. The sol was maintained at pH 3.5 with subsequent stirring for 24 h. The sol-gel reaction of the boehmite showed hydrolysis and pol condensation, shown in Figure S1. The detailed procedure was described in our previous study [22].

**Figure 1:**
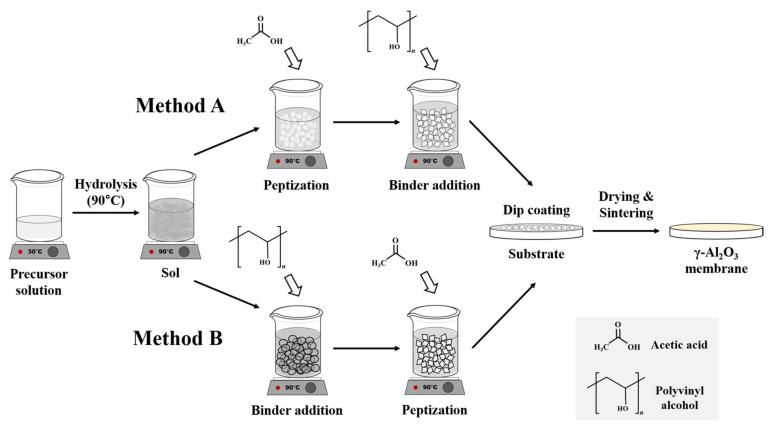
Schematic diagram for the preparation of γ-Al_2_O_3_ ultrafiltration (UF) membrane using the sol-gel methods A and B.

A 10 wt.% aqueous solution of PVA (M.W. 31,000–50,000, Sigma Aldrich, Darmstadt, Germany) was prepared in DI water. This PVA solution was incorporated into the boehmite sol using two different approaches. In the conventional approach (method A), the PVA was introduced into the boehmite sol after peptization, whereas in the modified approach (method B) it was added before peptization, as shown in Figure 1. The final membrane layers synthesized by methods A and B are denoted as membranes A and B, respectively. A γ-Al_2_O_3_ membrane without the PVA additive was also prepared as a reference (membrane S).

The substrate material used for the preliminary experiments was a disk-shaped α-Al_2_O_3_ porous support with a diameter of 47 mm. In the final stage, a multi-channel α-Al_2_O_3_ porous support with cylindrical tube-shaped dimensions (outer diameter: 24 mm, length: 150 mm) and 30 inner holes (inner diameter: 2 mm) prepared in our laboratory was used for the synthesis of the active γ-Al_2_O_3_ membrane layer [27]. The average pore size of macroporous support and top microfiltration layer was reported to be 0.8 μm and 0.07 μm, respectively. γ-Al_2_O_3_ membranes were formed on the inner channels of the tubular substrate by dip-coating with a soaking time of 50 s. Finally, the coated substrates were dried at ambient temperature for 24 h and sintered at 600 °C (1 °C/min) for 3 h.

### 2.2. Characterization

A low-voltage scanning electron microscope (Merlin Compact, Carl Zeiss, Jena, Germany) was used to observe the microstructure of the membrane and the thickness of the coatings. A ViscoQC™ 100-L viscometer (Anton Paar, Graz, Austria) was used to measure the viscosity of the boehmite sol. The specific surface area and pore size of the unsupported membranes were measured using the BET method and a surface area and porosity analyzer (BELSORP-mini II, MicrotracBEL, Osaka, Japan). The membrane porosity was determined using the gravimetric method [28]. Membranes were immersed in hot water for 3 h before measuring their wet weight ($m_{2}$). The dry weight ($m_{1}$) of the membranes was measured by placing them in an oven for 24 h. The membrane porosity (ε) was calculated using Equation (1):

(1) $$\varepsilon =\frac{\left(m_{2}-m_{1}\right)\rho_{1}}{\rho_{1}m_{2}+\left(\rho_{2}-\rho_{1}\right)m_{1}}$$

where $\rho_{1}$ and $\rho_{2}$ are the densities of Al_2_O_3_ (3.95 g/cm^3^) and water (1 g/cm^3^), respectively.

The filtration performance of the γ-Al_2_O_3_ membranes was determined by measuring the pure water (PW) permeability using a dead-end filtration system. The measurements were carried out at a transmembrane pressure of 2 bar, and the permeability was reported in units of LMH/bar (L·m^−2^·h^−1^·bar^−1^). The MWCO values were determined employing polyethylene glycol (PEG, Sigma-Aldrich, St. Louis, MO, U.S.A) retention experiments at six different molecular weights (1000, 1500, 3000, 6000, 10,000, and 20,000 g/mol) using a total organic carbon (TOC) analyzer (TOC-V CPH, Shimadzu Corp., Kyoto, Japan). The molecular mass of PEG corresponding to 90% retention was determined as the MWCO of the membrane. Additionally, regression analysis was performed using the extreme model to study the relationship between the rejection coefficient and the solute diameter [29].

## 3. Results

### 3.1. Effect of PVA Addition on the Thickness of γ-Al_2_O_3_ Membranes

**Figure 2:**
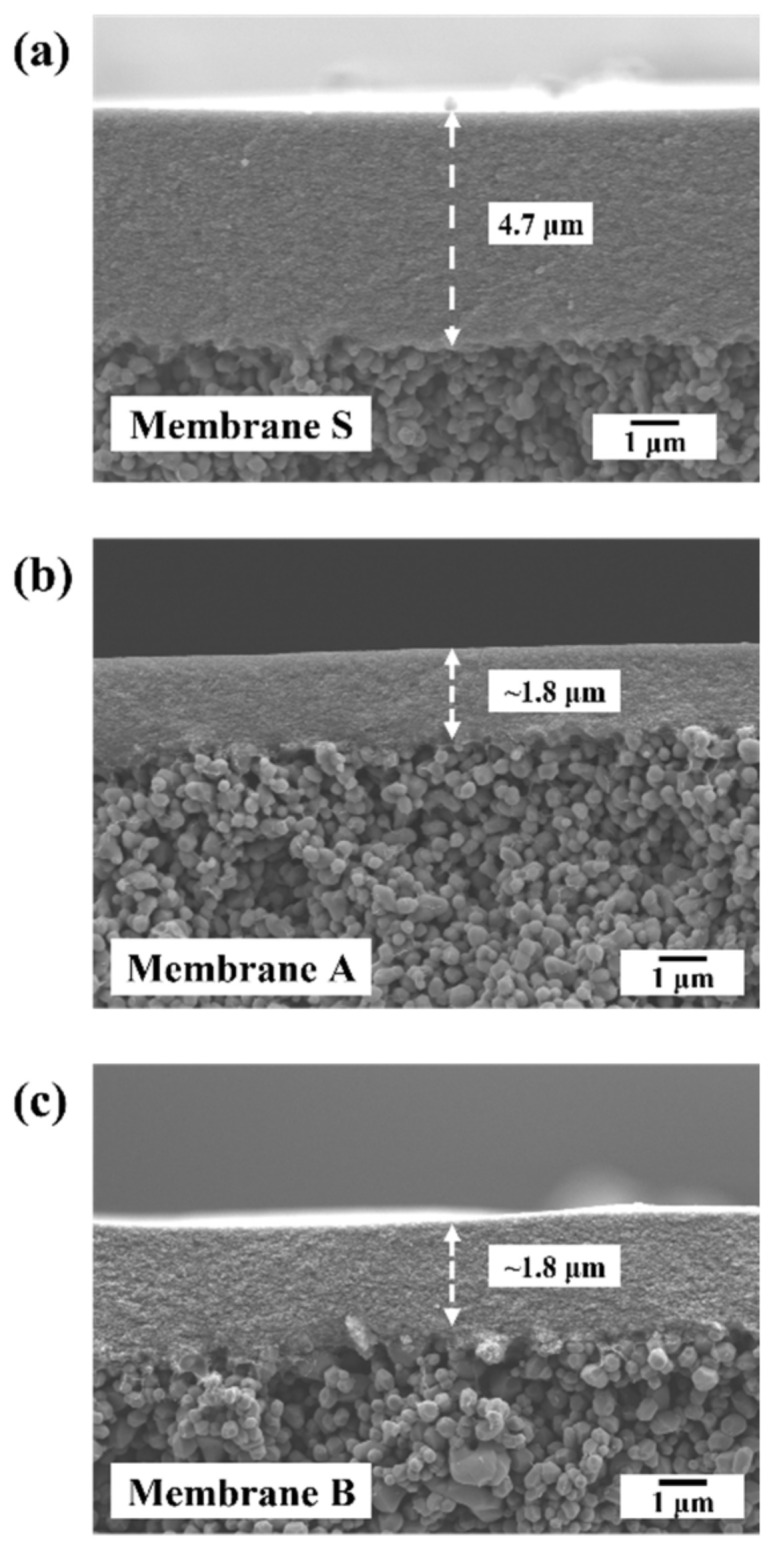
Top surface SEM micrographs of γ-Al_2_O_3_ tight ultrafiltration (UF) membranes: (**a**) membrane S (without the addition of PVA); (**b**) membrane A (addition of PVA after peptization); and (**c**) membrane B (addition of PVA before peptization).

Figure 2 presents the cross-sectional micrographs of the prepared γ-Al_2_O_3_ membranes. The resulting membranes were well-integrated with the substrate surface. The intermediate microfiltration layer and the top γ-Al_2_O_3_ UF layer are easily distinguishable. Membrane S had an average thickness of 4.7 μm, while the reported average thickness of membranes A and B was approximately 1.8 μm. With the addition of PVA, the thickness of the membrane layer decreased by half.

Gu et al. [30] described the formation of a membrane layer on a porous substrate using a mathematical model. The detailed parameters of the model can be found in equation (S1).

According to the proposed model, when the same substrate and dip coating conditions are used, the thickness of the membrane is directly proportional to the surface tension and inversely related to the viscosity of the suspension. To demonstrate the proposed decrease in the membrane thickness with increasing PVA concentration, the model must be validated.

**Figure 3:**
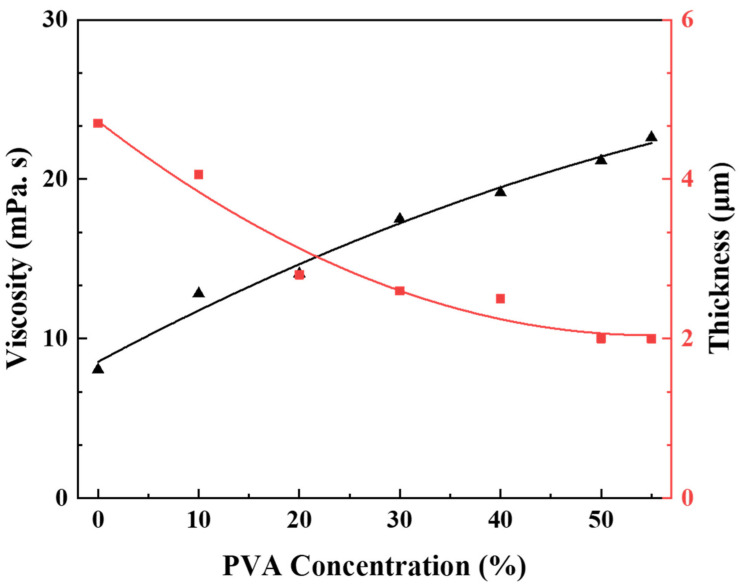
Schematic presentation of solution viscosity and thickness of γ-Al_2_O_3_ membrane A as a function of the PVA concentration in boehmite sol.

To this end, a boehmite sol was prepared with increasing PVA concentration ranging from 0 wt.% to 55 wt.%. The viscosity of the sol was determined by using a viscometer. The thickness of the top membrane layer was monitored using scanning electron microscopy (SEM). The effect of the sol viscosity on the thickness of the γ-Al_2_O_3_ membrane is illustrated in Figure 3. The viscosity of the solution increased with PVA concentration, whereas the thickness of the membrane layer on the α-Al_2_O_3_ substrate surface decreased. Moreover, the addition of PVA decreased the surface tension of the solution [31,32,33]. Consequently, adding PVA to the boehmite sol likely reduced the surface tension of the boehmite chains. According to the model, the membrane thickness tends to decrease with increasing viscosity and decreasing surface tension. Therefore, these results support the presented model and explain the decreasing trend of the membrane thickness as a function of the increasing PVA concentration in the boehmite sol.

Generally, the coating layer thickness should be as small as possible without any defects to obtain higher permeate flux [30]. As shown in Figure 3, the minimum thickness of the γ-Al_2_O_3_ membranes occurred at a PVA concentration of 50 wt.%. There was no significant improvement in membrane thickness with higher PVA concentrations. Additionally, the surface area of the membrane increased with the increasing concentration of PVA, as shown in Figure S2. Both minimum thickness and greater surface area favored the optimization of PVA at a concentration of 50 wt.% for further investigation.

### 3.2. Influence of the Fabrication Routes on the Properties of γ-Al_2_O_3_ Membranes

**Figure 4:**
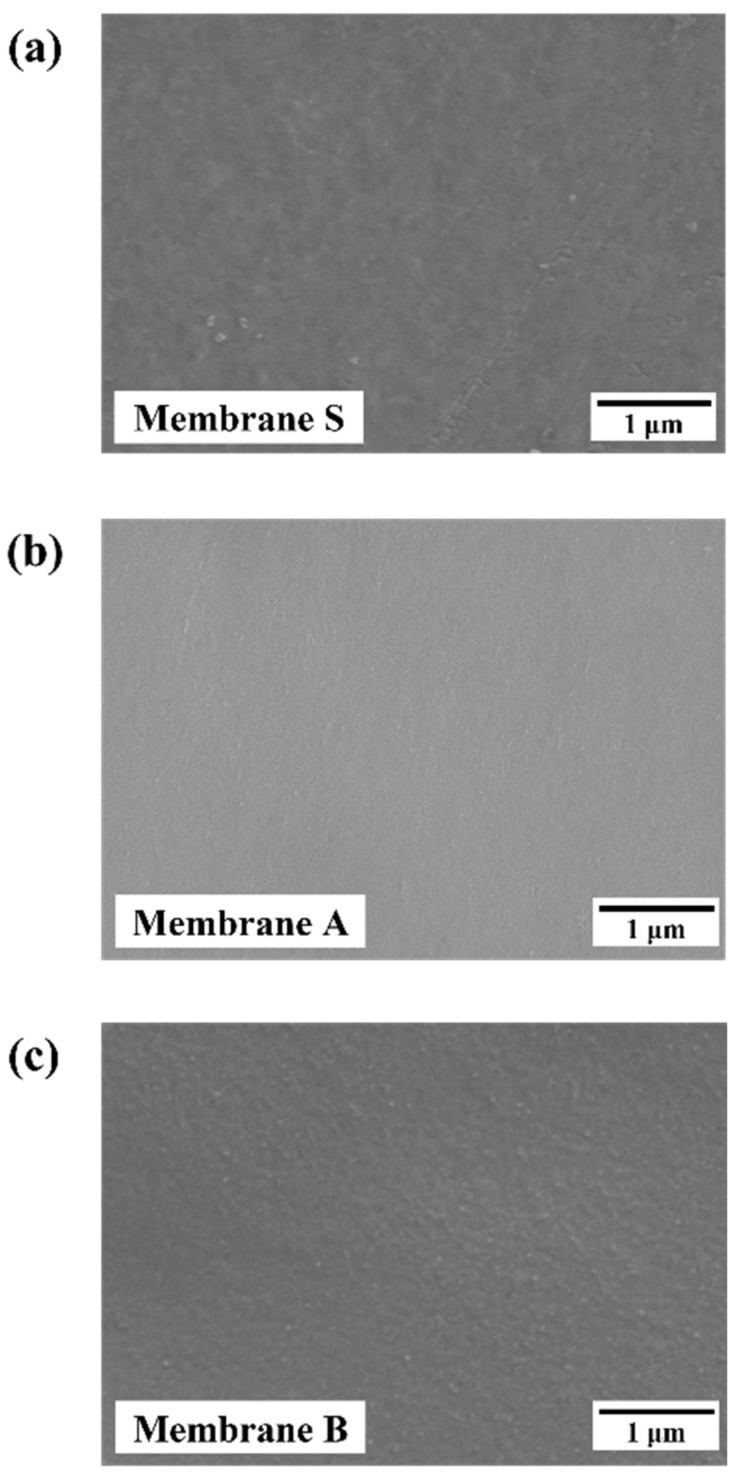
Top-surface SEM micrographs of γ-Al_2_O_3_ tight ultrafiltration (UF) membranes: (**a**) membrane S (without the addition of PVA); (**b**) membrane A (addition of PVA after peptization); and (**c**) membrane B (addition of PVA before peptization).

In the previous section, we discussed the effect of PVA addition on the thickness of γ-Al_2_O_3_ membranes. In this section, we investigate the influence of the fabrication route on the properties of the γ-Al_2_O_3_ membranes. Figure 4 shows the SEM micrographs of the top surface of the membranes prepared with and without PVA addition. Crack-free membranes with homogenous surface morphologies were obtained.

Nitrogen adsorption experiments were conducted to determine the pore characteristics of the prepared γ-Al_2_O_3_ membranes. The quantitative nitrogen sorption data are reported in Table 2.

Membrane S exhibited the smallest surface area and pore volume. On the other hand, membranes A and B demonstrated different pore characteristics, despite the similar PVA content. Furthermore, membrane B exhibited the highest surface area and pore volume. However, the average pore size of membrane B was approximately 7.4 nm, which was slightly larger than that of membrane A (7.0 nm). The addition of PVA increased the porosity of the γ-Al_2_O_3_ membrane (membrane S → membrane A), as shown in Table 3. Modifying the sequence of PVA addition resulted in a further increase in porosity, from 37.3 to 40.8% (membrane A → membrane B).

It is evident from the data that incorporating PVA before the peptization reaction significantly improves the morphological properties of the γ-Al_2_O_3_ membranes during sol-gel synthesis. The effect of the modified sol-gel method B on the surface area and porosity can be explained by examining the interaction mechanism between the PVA and the boehmite sol.

**Figure 5:**
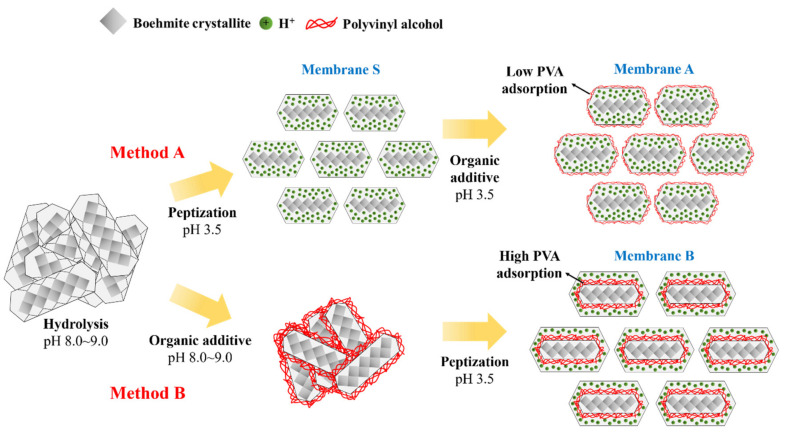
Schematic diagram of the proposed mechanism for the formation of γ-Al_2_O_3_ tight ultrafiltration (UF) membranes using methods A and B.

First, it is important to consider the chemical nature of PVA. PVA contains a large number of intermolecular and intramolecular hydrogen bonds. When it is added to a suspension, it forms a hydrogen bond with the hydroxyl groups on the surface of the boehmite particles [34,35]. Moreover, commercial PVA is not completely hydrolyzed and contains charged groups. PVA (hydrolysis degree 98%) contains 2% of unhydrolyzed chains of acetate groups (CH_3_COO^-^) [36]. These negatively charged groups allow PVA to interact electrostatically with the solid particles. Thus, the adsorption of PVA on the surface of the boehmite particles is governed by both hydrogen bonding and electrostatic forces. The proposed interaction of PVA during the synthesis of γ-Al_2_O_3_ membranes using methods A and B is illustrated in Figure 5.

The initial step of method A involved adding acetic acid as a peptizing agent after the hydrolysis reaction, resulting in the synthesis of membrane S. The pH of the hydrolyzed sol was in the range of 8.0–9.0, but it decreased with higher acetic acid concentrations. The boehmite sol was optimized at a pH of 3.5, which was much lower than the isoelectric point (pH 7.2–8.2) [37]. As previously reported [22], acetic acid dissociates into anions (CH_3_COO^−^) and cations (H^+^) that adsorb on the surface of boehmite particles. As a result, electrostatic repulsive forces are generated among the particles that stabilize the colloidal suspension and form a defect-free membrane.

In the following step, we introduced PVA as an organic additive in the peptized boehmite sol for the preparation of membrane A. As previously discussed, PVA interacts with boehmite particles via hydrogen bonding and electrostatic interactions. However, the presence of acetic acid ions on the surface of boehmite particles inhibits the reaction and adsorption of PVA molecules. As a result, a thin PVA layer adsorbed on the surface of the boehmite particles. As the amount of adsorbed PVA decreases, its pore-forming effect is compromised. The BET results support the proposed effects, demonstrating that the pore size of membrane A was smaller with a lower surface area compared to membrane B. This indicates that adding PVA does not significantly improve the morphological properties of the synthesized membrane using method A.

In the modified method B, PVA was introduced as an organic additive prior to the peptization reaction. This allowed PVA to interact with boehmite particles without the interference of acetic acid. It is expected that a larger number of PVA molecules can be adsorbed on the solid surface, leading to the enhancement of the pore-forming effect during the calcination process. This explains the slightly larger pore size of the modified γ-Al_2_O_3_ membrane, and the higher surface area and porosity compared to the conventional membrane A.

**Figure 6:**
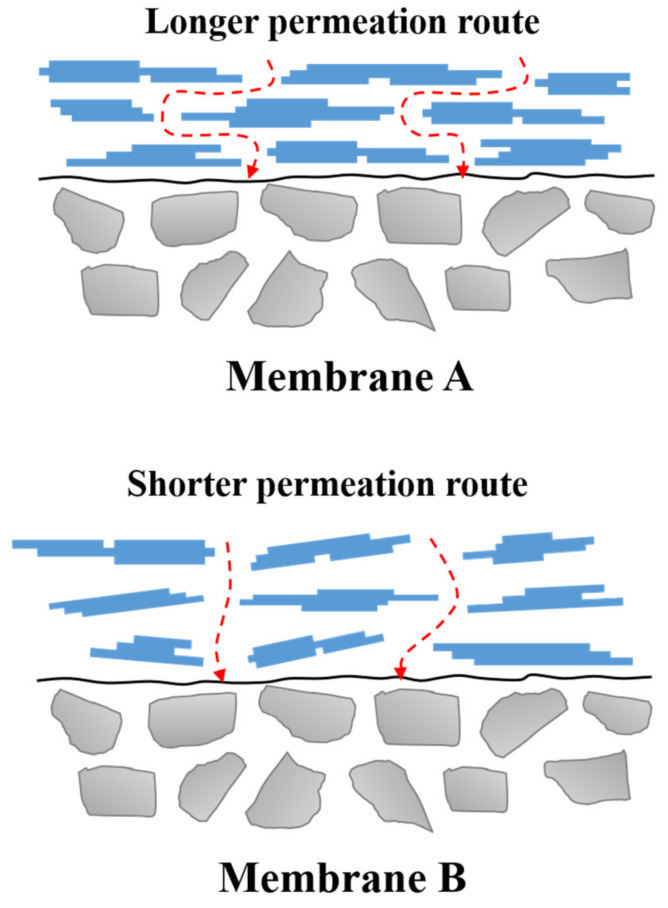
Schematic diagram of the possible permeation routes for membrane A and membrane B.

The structural differences between membranes A and B also affect their tortuosity. The tortuosity (τ) depends on porosity, particle shape and connectivity. Figure 6 shows the effect of tortuosity according to the pore characteristics of the sintered membranes A and B. Since the porosity of membrane B is higher than that of membrane A, membrane B has a lower tortuosity. Many attempts have been made to derive a correlation between tortuosity and porosity by introducing parameters representing the membrane pore structure [38]. The tortuosity has been reported to be inversely proportional to the porosity. As the porosity increases, the tortuosity decreases and converges to the ideal value of 1. Representative correlations between the tortuosity and the porosity are as follows [39]:

(2) $$\tau =\frac{{\left(2-\varepsilon \right)}^{2}}{\varepsilon }$$

(3) $$\tau =\frac{1}{\varepsilon }$$

Equations (2) and (3) were derived from the geometry of interstices between closed packed spheres and loose packed spheres, respectively.

According to Darcy’s law [40], high porosity and surface area, and low tortuosity contribute to the synthesis of γ-Al_2_O_3_ membranes with a high PW flux. This can be validated by experimentally determining the permeate flux of the membrane and comparing it to the mathematical model, as described in the next section.

### 3.3. Performance of the γ-Al_2_O_3_ Membranes

**Figure 7:**
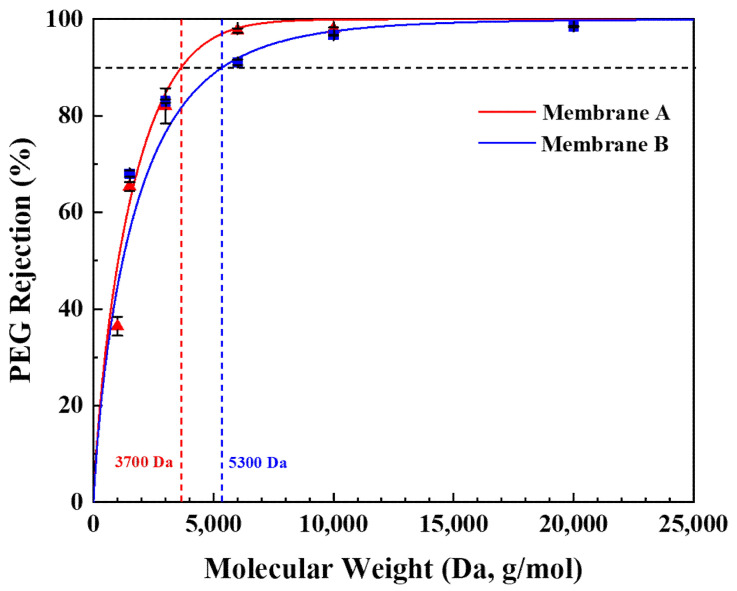
PEG retention curves of the γ-Al_2_O_3_ membrane A and B.

Figure 7 shows the PEG retention curves for the γ-Al_2_O_3_ membranes. The MWCO of membranes A and B were 3700 Da and 5300 Da, respectively. The corresponding Stokes diameter of the PEG molecules was calculated using Formula (4) [41].

(4) $$d_{s}=0.065\;{\left(\mathrm{MW}\right)}^{0.438}$$

Membrane A exhibited a pore diameter of 2.38 nm, while the calculated pore diameter of membrane B was 2.78 nm. Based on these results, both γ-Al_2_O_3_ membranes can be characterized as tight UF membranes with pore sizes in the mesoporous range. The difference in pore size between the membranes is attributed to the proposed effect of PVA adsorption on the surface of the boehmite particles.

**Figure 8:**
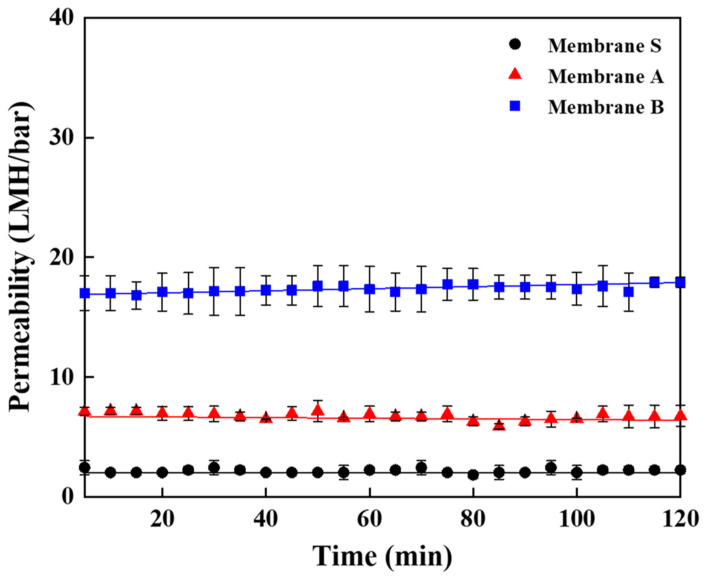
Pure water permeability curves by using the dead-end filtration system for γ-Al_2_O_3_ tight ultrafiltration (UF) membrane S, A and B.

A PW permeability test was performed using a dead-end filtration system. The permeability curves are presented in Figure 8. Membrane S exhibited the lowest permeability among the membranes. The PW permeability increased from 2.4 LMH/bar to 6.1 LMH/bar, with the addition of PVA during the synthesis of boehmite sol. The permeability of membrane B was 18.2 LMH/bar, which is three times higher than that of membrane A (6.1 LMH/bar). The variation of PW permeability was in agreement with the results of the porosity and surface area. A comparison of membranes A and B showed that the former had a relatively smaller surface area and porosity than the latter, and the latter had a higher permeability than the former. This indicates that the permeate flux increased with the porosity and surface area.

The effects of membrane thickness, pore size, and porosity on the permeate flux can be validated by the Hagen–Poiseuille model. The model was described in detail by Equation (S2) [42].

As per the model, the membrane thickness, pore size, and porosity play a significant role in the calculation of permeate flux. The thickness of the membrane can be minimized using PVA as a binder. The permeability of membranes A and B was significantly higher than that of membrane S. In addition, membrane B exhibited higher permeability because of its larger pore size and porosity compared to membrane A. 

We also compared our results with those of γ-Al_2_O_3_ membranes reported in the literature (Table 1). The γ-Al_2_O_3_ membrane synthesized by the modified method B in this work exhibits a much higher pure water permeability than the γ-Al_2_O_3_ membranes prepared by the conventional method in the literature.

**Figure 9:**
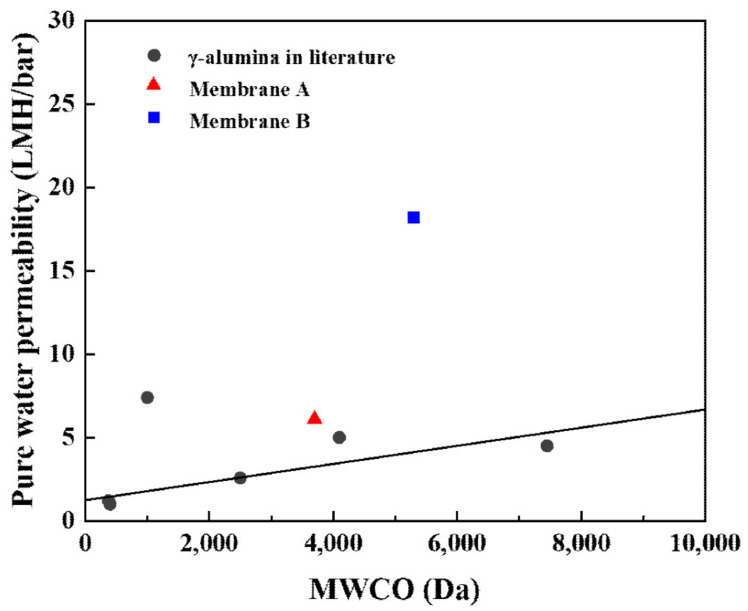
Correlation between MWCO values and pure water permeability of different γ-Al_2_O_3_ tight ultrafiltration (UF) membranes reported in the literature.

Figure 9 shows the correlation between MWCO values and the PW permeability of γ-Al_2_O_3_ ultrafiltration (UF) membranes reported in the literature [11,12,16,20,43,44]. It is evident that membrane thickness, porosity, and surface area play important roles in determining the permeate flux.

**Figure 10:**
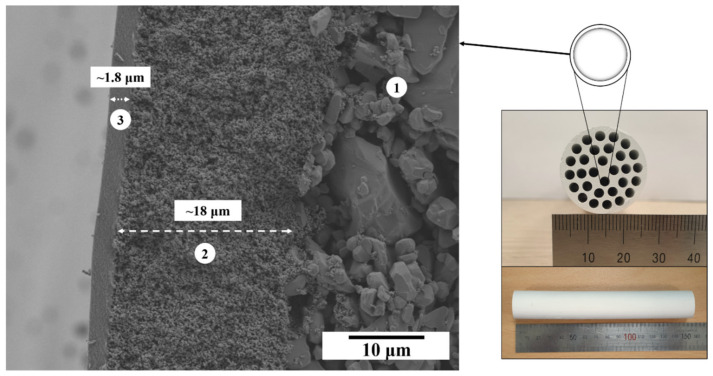
Cross-section of the asymmetric multichannel cylindrical tube-type substrate: (1) macroporous support; (2) intermediate microfiltration layer; and (3) γ-Al_2_O_3_ tight ultrafiltration (UF) layer.

Following the high filtration efficiency of γ-Al_2_O_3_ membranes synthesized using the modified method B, we successfully developed it on a multichannel cylindrical tube-type support. Figure 10 shows a well-integrated active membrane layer with uniform thickness along the substrate. The thickness of the γ-Al_2_O_3_ membrane coated on a multichannel cylindrical tube-type substrate was in accordance with the membrane thickness observed for a disk-type substrate. This demonstrates the potential of the modified γ-Al_2_O_3_ membrane for industrial applications with high performance.

## 4. Conclusions

The mesoporous γ-Al_2_O_3_ membrane was successfully prepared through a modified sol-gel route followed by a dip coating and sintering process. The properties of γ-Al_2_O_3_ membranes, with different concentrations of PVA from 0 to 50 wt.% and by changing the fabrication route, were investigated in detail.

The membrane optimized at a PVA concentration of 50 wt.% exhibited a minimum thickness of approximately 1.8 μm. The SEM micrographs revealed that the γ-Al_2_O_3_ membrane with PVA concentration of 50 wt.% exhibited minimum thickness. There was no difference in membrane thickness by increasing PVA concentration above 50 wt.%. The BET specific surface area also increased with increasing concentration of PVA.

Two sol-gel methods, referred to as methods A and B, were used to synthesize γ-Al_2_O_3_ membranes with optimized concentration of PVA. Method A is the conventional method of adding PVA after peptization, while method B is a modified route in which the order of PVA addition is altered. The analysis indicates that adding PVA before peptization during the modified method B leads to an increase in the porosity and surface area, and a decrease in tortuosity. This can be explained by the interaction between PVA macromolecules and boehmite particles without the interference of acetic acid ions, which enhanced the adsorption of PVA on the surface of the boehmite particles. The high adsorption facilitated the pore-forming effect of PVA.

Finally, a γ-Al_2_O_3_ membrane with a high pure water permeability of 18.2 LMH/bar was obtained using a modified sol-gel method. The addition of PVA in a modified synthesis approach proved to be effective in tuning the membrane morphological properties and increasing the PW permeability.

## Figures and Tables

**Table 1:** Comparison of the characteristics of γ-Al_2_O_3_ membranes from references.

Sintering Temperature	Specific Surface Area	Pore Size	Thickness	Permeability	Reference
(°C)	(m^2^/g)	(nm)	(μm)	(LMH/bar)	
500	-	4.4	-	4.5	[11]
600	271	5.5	~7	5	[12]
600	205	5.5	5.6	9	[13]
600	-	2.8	~1	1.1	[15]
500	-	1.9	1.5	5.4	[18]
600	260	2.7	~2	7.4	[20]
600	329.3	2.7	~1.8	18.2	Our work

**Table 2:** Pore characteristics determined by nitrogen adsorption experiment for γ-Al_2_O_3_ membranes.

	Sample		
	Membrane S	Membrane A	Membrane B
Specific surface area (m^2^/g)	226.17	293.84	329.36
Mean pore size (nm)	7.3329	7.0191	7.4434
Total pore volume (cm^3^/g)	0.4146	0.5413	0.6129

**Table 3:** The values of porosity of γ-Al_2_O_3_ membranes: membrane S (without the addition of PVA); membrane A (addition of PVA after peptization); and membrane B (addition of PVA before peptization).

Sample	Porosity (%)
Membrane S	29.2
Membrane A	37.3
Membrane B	40.8

## Data Availability

Not applicable.

## Supplementary Materials

The following supporting information can be downloaded at: https://www.mdpi.com/article/10.3390/membranes13040405/s1, Figure S1: Sol-gel reaction of the boehmite, (a) hydrolysis, and (b) poly-condensation; Figure S2: Schematic presentation of surface area of unsupported γ-Al_2_O_3_ membrane A and PVA concentration in boehmite sol.
