# Supplementary material for: High-Performance γ-Al2O3 Multichannel Tube-Type Tight Ultrafiltration Membrane Using a Modified Sol-Gel Method
Source: Membranes (Basel). 2023 Apr 3;13(4):405. doi: 10.3390/membranes13040405 (PMC10142786; doi:10.3390/membranes13040405)
Supplement: Supplementary file 1 [file membranes-13-00405-s001.zip › membranes-2290130-supplementary.pdf]

## S1. Chemical reaction of the boehmite sol

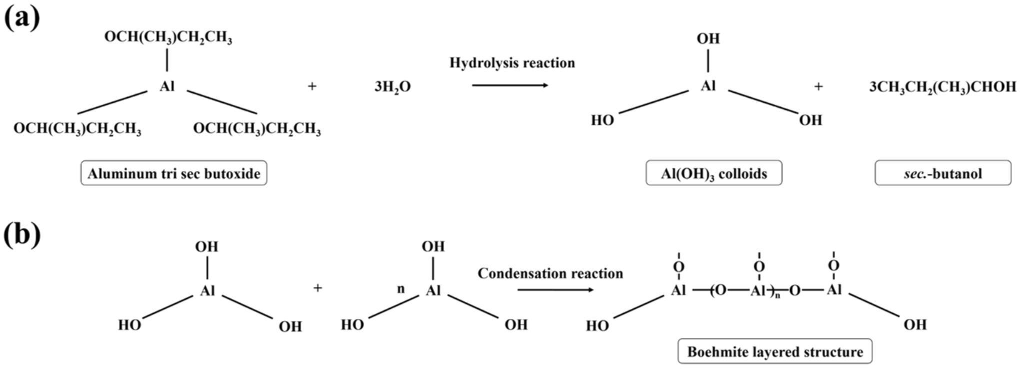


**Figure S1.** Sol-gel reaction of the boehmite, (a) hydrolysis, and (b) poly-condensation.

## S2. Formation of membrane layer

*S2.1 Mathematical model*

Gu et al.[1] described the formation of a membrane layer on a porous substrate using the following model (S1):

$$L_m = 2\left(\frac{\varepsilon_S \gamma K_m}{\eta \alpha R} t\right)^{1/2} \quad \text{(S1)}$$

where $L_m$ is the membrane thickness, $\varepsilon_S$ is the porosity of the substrate, $\gamma$ is the surface tension of the liquid solution, $K_m$ is the permeability of the formed membrane, $\eta$ is the viscosity of the solution, $\alpha$ is the ratio of the particle concentration in the solution to that in the wet membrane, $R$ is the pore radius of the substrate, and $t$ is the dip-coating time.

*S2.2. Effect of PVA concentration on surface area*

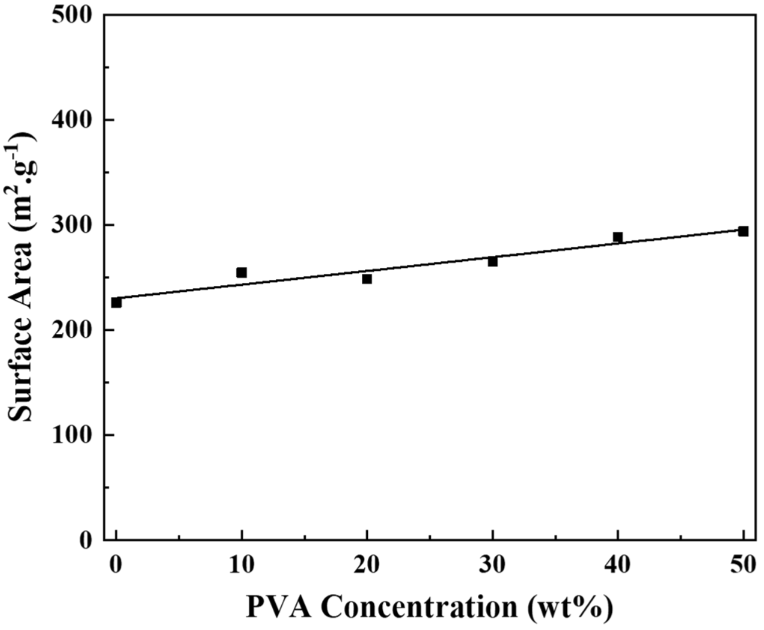


**Figure S2.** Schematic presentation of surface area of unsupported $\gamma$-$Al_2O_3$ membrane A and PVA concentration in boehmite sol.

## S3. Calculation of permeate flux

Hagen-Poiseuille model[2] described the effects of membrane thickness, pore size, and porosity on the permeate flux using the following equation (S2):

$$j = \frac{\Delta P {d_m}^2 \varepsilon}{32 \mu L \tau} \tag{S2}$$

where $j$ is the pure water flux, $\Delta P$ is the transmembrane pressure, $d_m$ is the pore diameter, $\varepsilon$ is the membrane porosity, $\mu$ is the liquid viscosity, $L$ is the membrane thickness, and $\tau$ is the tortuosity of the membrane.

## References


[1] Y. Gu and G. Meng, "A model for ceramic membrane formation by dip-coating," *J. Eur. Ceram. Soc.*, vol. 19, no. 11, pp. 1961–1966, 1999, doi: 10.1016/s0955-2219(99)00013-8.

[2] P. Marchetti, A. Butté, and A. G. Livingston, "An improved phenomenological model for prediction of solvent permeation through ceramic NF and UF membranes," *J. Memb. Sci.*, vol. 415–416, no. December, pp. 444–458, 2012, doi: 10.1016/j.memsci.2012.05.030.
